# Automated systems — the users' needs from a manufacturer's point of view

**DOI:** 10.1155/S1463924681000187

**Published:** 1981

**Authors:** T. M. Craig

**Affiliations:** E.I. Du Pont de Nemours Clinical Systems Division Wilmington Delaware USA


### Automated systems – the users' needs from a manufacturer's point of view*

T.M. Craig
*E.I. Du Pont de Nemours, Clinical Systems Division, Wilmington, Delaware, USA.*

### Introduction

Successful development and marketing of an automated product requires accurate identification of the needs that the instrument can satisfy and the expression of those needs as parameters for product design. Once these parameters are set the manufacturer must develop a product prototype to satisfy them. Finally, extensive field testing of the prototype must be carried out before making the instrument commercially available. This is very critical, especially in healthcare automation.

### The Du Pont 'aca' analyser

The development of the Du Pont 'aca', a clinical analyser that automates 42 tests on blood serum and body fluids, illustrates the above process. The heart of the 'aca' system is the analytical test pack containing prepackaged reagents in their stable form. On top of the pack is a plastic header. This carries a binary code which enables the instrument to identify the test being run. On the header is also imprinted the name of the test. The plastic pack which also serves as a test cuvette in which the chemical analysis takes place is shown in Figure 1.

The concept of the 'aca' is straightforward. An unmeasured amount of serum or other body fluid is poured into a sample cup. A patient identification card is filled out (for later transfer by photographic imaging to the test report slip) and put into the instrument along with the cup. The test packs, representing the tests requested by the physician, are then loaded into the 'aca' behind the sample cup. After the instrument is activated the first result appears in seven minutes and thereafter every 37 - 74 seconds. The test report appears with the initial = information recorded on the patient and a digital print of the results of the tests processed on that particular patient. The system design is shown in Figure 2.

### User requirements

In developing the design parameters for the 'aca' Du Pont based its decisions on user requirements; this paper will use as an example those of STAT or emergency testing which is the most demanding application of automation. These requirements were ranked in priority order as assessed by the development team after extensive discussions with clinical laboratory personnel.

(1) *Quick turn-round time.* STAT testing demands quick results. The plan therefore was to develop a system that would provide the first test results in less than eight minutes and successive test results every 37 to 74 seconds.

(2) *Round-the-clock availability.* The second requirement in STAT testing is that the automated system be available 24 hours a day, 7 days a week. The design parameter was that the 'aca' always be on standby when not in use with no start-up or shut-down time required before analyses can be commenced.

(3) *Minimum staffing.* It is difficult to have skilled technologists available at all times, particularly at night and weekends. Therefore, the third user requirement is for a system that is relatively easy to use. The matching design parameter of the 'aca' was simplicity of operation with minimum operator involvement.

(4) *Accuracy and precision.* Because STAT tests are often the most critical tests the fourth user requirement is a high degree of accuracy and precision. The design parameter set was that a coeficient of variation equal to or less than 5 percent on enzyme tests and equal to or less than 3 percent on chemical tests should be obtained in routine use. Du Pont also provides the use of reference methodology in chemistry procedure development.

*Paper presented at Analysis 79 Symposium – Automation in Industrial & Clinical Chemistry, London, UK, July 1979. This paper complements the views expressed by Dr. Bierens de Haan at the same conference when he discussed the clinical chemist's views on instrument design requirements. This latter paper was published in the* The Journal of Automatic Chemistry, *Vol. 2, No. 2, pp. 57, 1980.*

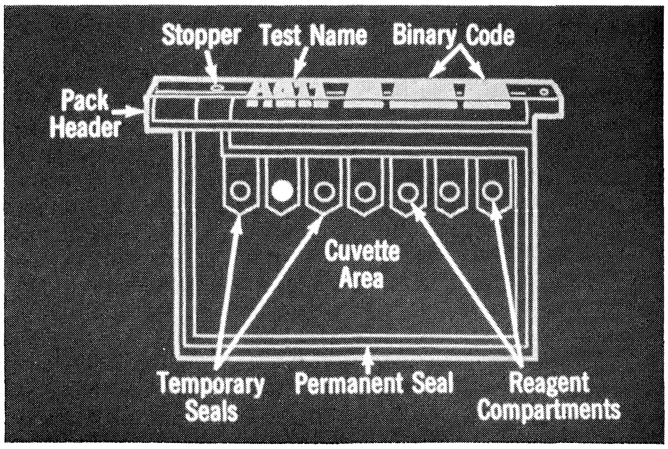

*Figure 1.*

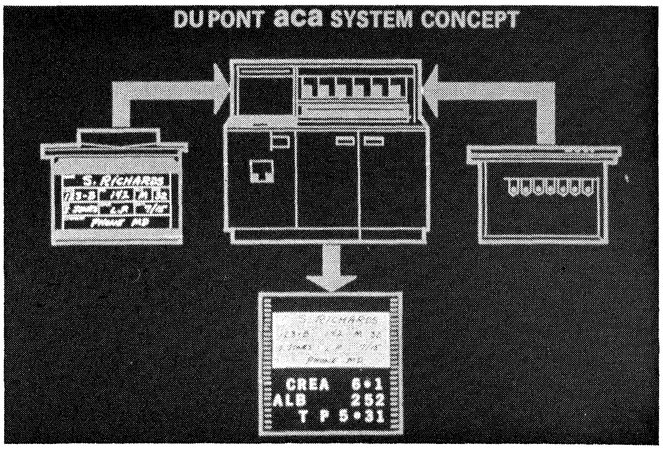

*Figure 2.*

(5) *Compatibility with routine procedures.* Another STAT requirement is that the test results should be compatible with routine procedures. Often hospitals, particularly medium and large ones, have several clinical analysers one of which may run the bulk of routine chemistries during the day. In this situation the 'aca' often complements the other analyser by running the STAT and speciality tests during the days, nights and weekends. It is very important that physicians see only one set of normal ranges in order to reduce excessive interpretation. Thus, the 'aca' design parameter was that the slope and the intercept should be adjustable. The user laboratory can then run split sample comparison studies and develop one set of normal ranges for those tests that are run on more than one system.

(6) *Positive patient identification.* The sixth critical requirement is positive patient identification; no mistaking of the patient sample and test result must be possible. The corresponding design parameter for the 'aca' was photographic transfer of patient information to the test report sheet.

(7) *Variety of tests.* The seventh market requirement is for a variety of automated tests. The 'aca' was introduced in 1971 with eight automated tests. Currently the 'aca' performs 42. Virtually all tests that are carried out in the STAT mode in the clinical chemistry area have been automated on the 'aca'. In addition, one or more tests may be requested on a particular patient by the physician, so the system must operate in a discrete mode with zero changeover time between the different test procedures.

Identifying these priorities is an important product development process. The manufacturer identifies the requirements of the marketplace, sets priorities for these, and then develops design criteria so that the product will fit the designated applications.

### Field evaluations

Once a manufacturer develops the product prototype, the next stage is to test it in the field. It is important that extensive field evaluations be conducted by capable and demanding potential users prior to marketing.

In the case of the 'aca', three major field evaluations were conducted in the USA – the University of Wisconsin Medical Center, the Upstate Medical Center in Syracuse, New York, and the University of Alabama Medical Center. As a result of these evaluations, a monograph was published and widely distributed by those involved in the field evaluation at the University of Alabama [1]. A paper by the evaluators at the Upstate Medical Centre in Syracuse was published [2]. Therefore, the information from the evaluations was widely disseminated for study by clinical laboratory personnel.

Although field evaluation is very expensive and time consuming, it is vital. It is absolutely imperative that the manufacturer does these evaulations so that the buyer of the equipment does not have to. The advantage from the buyer's standpoint is that the system has been thoroughly evaluated, so it can be rapidly put into routine clinical use.

Field evaluation is also critical from the standpoint of the manufacturer. Earlier users will provide a satisfied referral base which is a major factor in the success of a new product.

### Requirements upon marketing

Once the product has been launched commercially, the manufacturer must address a different set of requirements and spend considerable resources to satisfy them. In clinical laboratory automation, the first of these needs is service. Using the Du Pont 'aca' as an example, the most stringent service requirements that apply to STAT market service were matched against the programme parameters of manufacture; these are discussed below.

(1) *Instrument reliability.* The first service requirement is for minimum downtime, which means a manufacturer must place a high priority on instrument reliability. Du Pont studies show that the 'aca' system is operative about 98 percent of the time. Du Pont also has a retrofitting programme for the 'aca'. Developments in the design of the 'aca' that improve reliability are retrofitted to all instruments in the field. By continuously improving the instrument's reliability, the downtime is further minimised.

(2) *Round-the-clock servicing.* In addition, a very extensive and expensive servicing programme ensures minimum downtime in STAT testing automation. Because the 'aca' system operates 24 hours a day, 7 days a week, around-the-clock service is required. This is handled through a large network of satellite service engineers, backed up by regional and area service centres.

(3) *Modular components.* The 'aca' system design is modular so that parts can be easily replaced. The service representative doesn't have to fix the part on site but, rather, can interchange the necessary part to get the system back in operation quickly. To ensure that parts are available when needed, Du Pont has set up regional parts depots at several major airports. Once a service engineer has contacted a laboratory, identified the problem and isolated it to one component, a call to the regional parts depot will have the necessary component on the next plane, thus ensuring minimum downtime.

(4) *Telephone trouble-shooting.* Telephone trouble-shooting is another very important aspect of Du Pont's total service programme. Every new user attends an 'aca' training session. With this background and the use of the operator's manual, trouble-shooting solves 9 out of every 10 problems over the phone, taking far less time than calling in a service engineer.

(5) *Preventive maintenance.* Du Pont schedules annually a programme of preventive maintenance calls for each location. Service representatives check out the system thoroughly and replace any defective or potentially defective components. At that time, any additional improvements developed since the last visit are retrofitted to the 'aca'.

### Training programme

Another support requirement once marketing is achieved is training. One reason is that clinical laboratories have high personnel turnover. In addition, a large number of medical technologists operate the system; not only during the daytime but at night and on weekends. Du Pont, therefore, trains thousands of people a year to operate the 'aca'.

This is accomplished through a comprehensive training programme. First, Du Pont has training centres throughout the world where systems are in routine clinical use: Wilmington, Delaware; Geneva, Switzerland; Claremont, California and Tokyo, Japan. There is a basic course, and an advanced one for those who have gone through initial training and operated the system.

Du Pont also has an audio-visual instruction series, that laboratories can use in-house. Also, Du Pont technical representatives and service engineers conduct user conferences with customers to discuss many aspects of the instrument and its use, for example calibration, quality control and trouble-shooting. A Du Pont technical representative also may go into a laboratory and carry out training on a one-on-one basis to develop personnel for required levels of expertise.

Very important to the training programme are readable manuals. The 'aca' manuals have been tested and are easy to follow; they are used as part of the training process and to answer questions. There is little likelihood they simply sit on the shelf.

### Staying up to date

Another area that is extremely important in developing a commercial product is the manufacturer's obligation to maintain the state-of-the-art technology for the user. This is especially critical in clinical laboratory automation, which is a fast-changing, high-technology area.

In the 'aca', this philosophy governs the technical advances in both the instrumentation and test chemistries. In 1971, the 'aca' I was introduced with a 30-channel capacity. Eight automated tests were available. Today a number of models and 42 tests are available. The 'aca' II has 60 channels, which can automate up to 60 tests. The 'aca' III is a microprocessor-controlled system with virtually unlimited test capabilities.

Many 'aca' users have upgraded their systems. Du Pont has attempted to make this upgrading as easy as possible by providing accessories that can be retrofitted onto the system. Whenever a new model or accessory is introduced, Du Pont takes steps to make it easy for existing users to increase systems capability. Therefore, a laboratory can, if it so elects, continue to use the same system it purchased initially but with the increased capability similar to the latest systems available to new users.

In some cases, it is not technically feasible to upgrade or retrofit a system; for example, going from an 'aca' I or an 'aca' II, which have solid state computer systems, to an 'aca' III, which is microprocessor controlled. In these cases, Du Pont offers an exchange programme which allows a fairly substantial trade-in value to minimise the cost of upgrading to existing 'aca' users.

In the chemistry area, Du Pont offers all new tests to every user.

A manufacturer also is responsible for providing various financial options for the customer because capital in the clinical laboratory is limited. In addition to purchase, Du Pont provides several leasing options and an instrument rental and a fee-per-test programme to fit customers' varying financial situations.

### Professional and ethical responsibilities

Manufacturers in the healthcare field also have other responsibilities.

One that Du Pont feels is particularly important is transfer and sharing of expertise. An example of this in the clinical laboratory is management training. Du Pont has seen this need develop for a number of years. It is not unusual for clinical laboratory technical personnel to move up to management training. Du Pont has developed a programme called "Insight", an eight-hour course on financial management in the clinical laboratory.

Supporting professional societies is another responsibility of manufacturers in the healthcare field. This support can be in terms of providing communications, legal or technical skills and monetary or human resources. For example, Du Pont personnel are actively involved in The US National Clinical Laboratories Standards Committee.

And lastly, the manufacturer in the clinical laboratory should provide ethical assistance in making an automation decision. Essentially, this is a process which gives key decision-makers information for judging whether or not automation will be of use to them.

For the 'aca', Du Pont conducts value-in-use analyses to determine the economic viability and cost effectiveness of the system, given the specific requirements of the individual clinical laboratory.

### Conclusion

In summary, a manufacturer of analytical instruments must first identify the requirements in the clinical laboratory and then develop the product that meets them. Second, the manufacturer must conduct extensive field evaluations of the product prior to marketing to ensure that the first purchasers can rapidly incorporate the product into routine clinical use.

Third, once a commercial instrument is available, the manufacturer must commit large resources to service, training and maintaining the state-of-the-art technology. Finally, the manufacturer must provide accurate representation and contributions to professional healthcare societies.

REFERENCES

[1] Perry, B.W., Hosty, T.A., Coker, J.J., Doumas, B. and Straumfjord, J.V., (1970) monograph published by Du Pont.

[2] Speicher, C.E., Fetrat, M.E., Fiske, M.L. and Henry, J.B., (1972) *American Journal of Clinical Pathology,* 57, (5) 643 - 658.

